# The role of soil in the contribution of food and feed

**DOI:** 10.1098/rstb.2020.0181

**Published:** 2021-09-27

**Authors:** W. L. Silver, T. Perez, A. Mayer, A. R. Jones

**Affiliations:** ^1^ Department of Environmental Science, Policy, and Management, University of California, Berkeley, CA 94720, USA; ^2^ Centro de Ciencias Atmosféricas y Biogeoquímica, IVIC, Caracas, Venezuela

**Keywords:** biogeochemistry, global food and feed, nature's contribution to people, soils, nitrogen, phosphorus

## Abstract

Soils play a critical role in the production of food and feed for a growing global population. Here, we review global patterns in soil characteristics, agricultural production and the fate of embedded soil nutrients. Nitrogen- and organic-rich soils supported the highest crop yields, yet the efficiency of nutrient utilization was concentrated in regions with lower crop productivity and lower rates of chemical fertilizer inputs. Globally, soil resources were concentrated in animal feed, resulting in large inefficiencies in nutrient utilization and losses from the food system. Intercontinental transport of soil-derived nutrients displaced millions of tonnes of nitrogen and phosphorus annually, much of which was ultimately concentrated in urban waste streams. Approximately 40% of the global agricultural land area was in small farms providing over 50% of the world's food and feed needs but yield gaps and economic constraints limit the ability to intensify production on these lands. To better use and protect soil resources in the global food system, policies and actions should encourage shifts to more nutrient-efficient diets, strategic intensification and technological improvement, restoration and maintenance of soil fertility and stability, and enhanced resilience in the face of global change.

This article is part of the theme issue ‘The role of soils in delivering Nature's Contributions to People’.

## 1. Introduction

In the most fundamental sense, soils are the basis for life on the Earth. Soils are the pedestal that plants rely on to remain erect. Soils provide the habitat for a vast biodiversity and biomass of soil organisms [1], and both generate and serve as the repository for most of the carbon (C) and nutrient elements that support life [2]. Soils retain the water that plants and soil organisms use to survive and grow, and slow the rate of water movement and thus limit the rate of erosion and soil loss [3]. Soils also contribute to the composition of the atmosphere, and by association impact climate, and are both a significant source and sink of greenhouse gases [4,5]. At a societal level, one of the most obvious contributions of soil to people is the role that soils play in the provision of food for human populations and feed for livestock. People have directly managed soils for food and feed production via agriculture for over 12 000 years [6]. For over 100 000 years, soils have been indirectly managed for food and feed production through hunting and gathering [7]. Approximately 40% of the terrestrial land surface is currently dedicated to food and feed production, with approximately 12% in crop agriculture and 25% in grazing lands [8].

Management for food and feed production in many ways is akin to mining. Plants extract nutrients from soils and store them in their tissues. Plant harvest transports soil nutrients out of the ecosystem, a process that is only one step removed when livestock convert plants to animal biomass that itself is harvested for human consumption. Nutrient-return to soils in the form of organic (plant, livestock and other organic matter resources) or inorganic fertilizers is critical to maintain soil fertility in agricultural soils. However, nutrient inputs rarely fully replace the nutrients extracted via harvest, leading to soil nutrient depletion and degradation over the long term [9–11].

Soils differ dramatically in the availability of the resources needed for food and feed production. For example, old, highly weathered soils typically contain low phosphorus (P) availability owing to the high P sorption capacity of residual iron and aluminium minerals, coupled with deep profiles and the lack of new P inputs from weathering products of primary minerals [12,13]. Inherent nitrogen (N) limitation is common in recently unglaciated regions and new, volcanically derived soils, as well as in high latitude regions with low mean annual temperature, or areas with little temperature seasonality, low mean annual precipitation, low soil clay fractions or high seasonality in precipitation [14]. The factors that control pedogenesis help determine the ability of soils to support agricultural productivity and the degree to which individual crop species may thrive.

The globalization of markets for agricultural products has led to the transport of soil nutrients from the site of origin to regions that differ greatly in geology, climate, biota and soil characteristics. Trade in agricultural products doubled between 1995 and 2018 [15]. Model estimates suggest that the movement of associated soil nutrients is extensive and increasing. For example, Grote *et al*. [16] predicted that the movement of N, P and potassium (K) doubled to 8.8 Tg between 1997 and 2020. Indeed, the movement of the four dominant staple crops (maize, wheat, rice and soy) increased 2.3 times between 1997 and 2019, suggesting that embedded nutrients likely increased at a similar rate [17]. Some of the soil nutrients tied up in agricultural products and transported via national and international trade will be immobilized in local biomass for years to decades. However, a proportion of the nutrients transported will end up in the waste stream within weeks to months after arrival. Approximately 194 to 389 kg y^−1^ of food is wasted per person globally [18], amounting to approximately one-third of food production [19]. Nutrients imported via food and feed that ultimately end up in the waste stream are rarely applied to depleted soils. Instead, these nutrients tend to concentrate in urban areas where they contribute to greenhouse gas emissions, eutrophication and other forms of pollution [16,20].

In this paper, we explore the role of soils in the provision of food and feed for a growing global population. We review regional patterns in soil characteristics in relation to agriculture and human nutrition. We use recent data on a subset of important agricultural products (maize, wheat, rice, soy and beef) and associated N and P concentrations to explore patterns in the production and transport of food, feed and associate nutrients via international trade. The role of soils in the provision of food and feed in local food systems is discussed. Finally, we discuss emerging challenges to agricultural soil systems and review proposed solutions for the future.

## 2. Food and feed for a growing global population

The global population is expected to increase by approximately 2 billion people by 2050 and reach almost 11 billion in 2100 [21]. Meeting the food demand for this growing global population is a major challenge of the twenty-first century. Increased crop production will be needed to supply sufficient food and feed over both the short and long terms [22]. This required increase in agricultural productivity is set against a backdrop of widespread and increasing land degradation [23,24] and growing challenges from a changing climate [25]. Some estimates predict that agricultural productivity will need to triple by the year 2100 to meet global demand under a business-as-usual scenario [26], with more agricultural production needed over the next 30 years than has been produced over the preceding 400 years [27]. Impending shortfalls in food-related calories and nutrients are exacerbated by growing inefficiencies in the use of soil resources. For example, meat and dairy consumption, increasing in wealthier nations, typically has lower efficiencies of conversion of crop calories into edible protein than consumption of non-meat staple crops [28,29]. Livestock remains an important source of food but is a drain on global food calories if fed with potential food crops. However, livestock is a net gain to the food system when fed with grass (aka pasture raised) or waste products that would not otherwise be used for human food [30]. For example, shifting 16 major crops to 100% food rather than animal feed would increase available food kilocalories by 49% globally [31]. A shift in calories from animal to plant sources may be counter to some cultural norms and present other barriers to adoption, but at a global scale could have a significant impact on meeting future food demand.

While food production will need to increase in all agricultural regions to meet growing demand, the predicted degree of food self-sufficiency varies greatly by region, with the lowest estimates in Africa and Southeast Asia. Deficiencies in these regions are owing in part to inherent soil and climatic conditions, as well as patterns in population growth that exceed projected increases in crop production using current approaches, together with socio-economic and political forces driving inequality in food and agricultural resource distribution [26,32].

## 3. The relationship of soils to feed and feed

### (a) The dominant soil types in global agriculture

**Figure 1.**
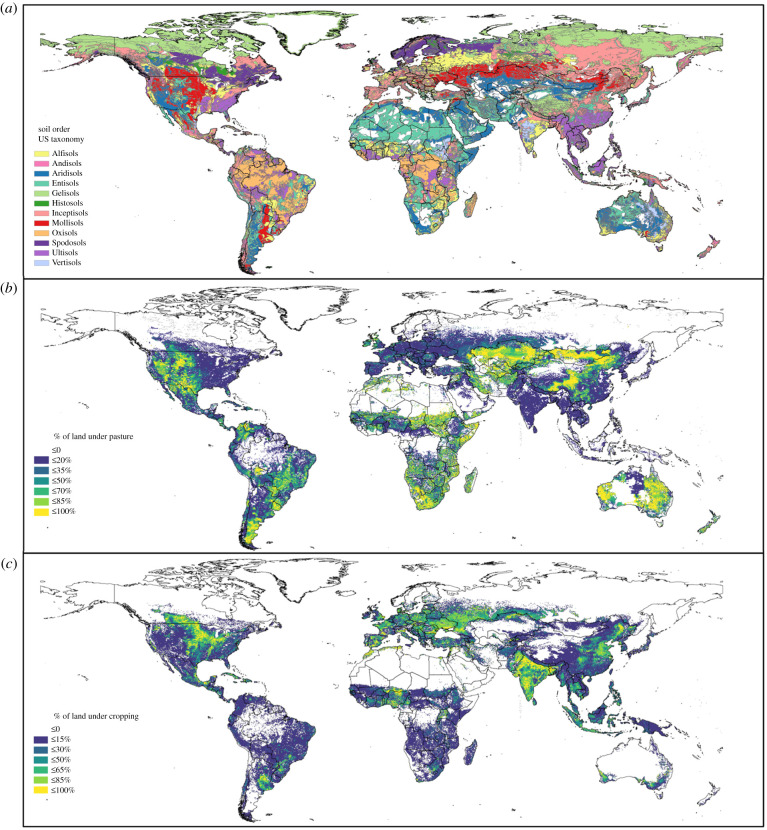
Global map of (*a*) soil order according to USDA taxonomy [34]; and proportion (%) of land under (*b*) pastures and (*c*) cropping in year 2000 [35,36]. (Online version in colour.)

From an ecological perspective, the potential productivity of agricultural land depends largely on the combination of inherent soil chemistry, physical characteristics and climate, all of which are encompassed in soil classification. Specific soil types tend to dominate global food and feed production when controlling for climate, owing to a set of physical and chemical properties that more strongly promote plant growth. Mollisols are among the most intensively farmed soils globally, particularly in the Americas, Europe and Asia (figure 1; [33]). Mollisols tend to be characterized by the accumulation of surface soil organic matter, which contributes to nutrient supply and to nutrient and water retention. Mollisols also tend to occur in stable landscapes lacking steep topographic change and thus are less affected by erosion than some other common soil orders [33,34]. Similarly, Alfisols in Africa and Europe, and some Inceptisols in Europe and south Asia are intensively farmed owing to inherently favourable nutrient availability derived from either high soil organic matter content or input of abundant weathering products [33,34]. Mollisols and Alfisols combined account for approximately 17% of the global land surface, while Inceptisols cover approximately 15% of the global land area (figure 1; [34]).

Entisols are generally characterized by shallow profiles, steep slopes and other easily erodible surfaces, and tend to support lower agricultural productivity in the absence of inputs, with the possible exception of Entisols developed from recent alluvial deposits [37]. Entisols cover almost 18% of the global land surface and are dominant in Africa and parts of Australia (figure 1; [34]). Aridisols (12% of the global land surface), or dry land soils, occur under generally non-arable conditions. These soils tend to host grazing activities where crops cannot grow (figure 1). Irrigation can facilitate plant growth in some Aridisols, but high salt content inhibits plant growth in others [38]. Aridisols in regions of North America, South Africa, Kazakhstan, Australia and Argentina are dominated by livestock grazing management (figure 1; [34,39]). Andisols, derived from recent volcanic activity, cover less than 1% of the Earth's land surface and can support particularly fertile soils. Deforestation for agricultural development is a growing concern on Andisols in tropical regions [40].

### (b) Agricultural soil nutrients

**Figure 2.**
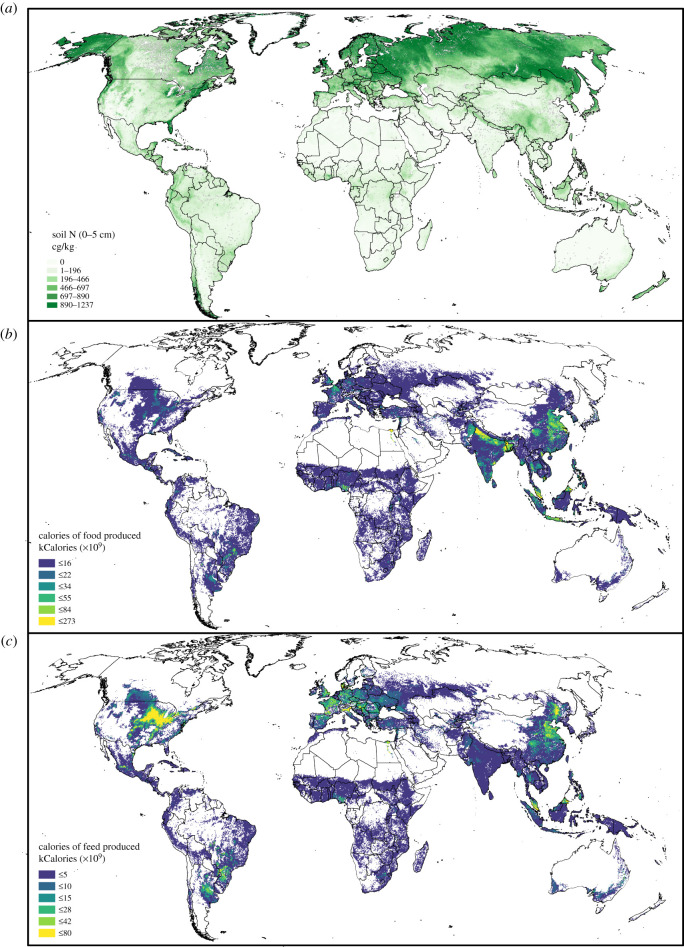
Global map of (*a*) soil N concentration in 0–5 cm depth (cg/kg; [42,43]), (*b*) production of food calories (kCalories; 1997–2003; [41]), and (*c*) production of feed calories (kCalories; 1997–2003; [41]).

Soils differ dramatically in their inherent nutrient availability. Much of the cropland with the highest native soil N contents in the world, which also have some of the highest crop yields, primarily produce livestock feed or biofuel (figure 2; [41,42]). In 2019, less than 10% of the calorie production in cropland in the USA was used for human consumption, with the majority of calorie losses owing to inefficiency in nutrient conversion of maize and soy to energy or animal meat through use as biofuel or livestock feed [44]. Despite lower overall soil nutrient availability and productivity, croplands in eastern and central sub-Saharan Africa and India have among the highest overall rate of conversion of crop-embedded nutrients and calories to human food globally. Crops contributing to high food calories include maize, wheat and cassava in southern Africa, sorghum, sugar crops, teff and roots and tubers grown in east Africa, and sugar crops, rice and legumes produced in India [17,41,45]. The remarkable influence of economics on croplands is apparent in Indonesia, which has some of the highest crop productivities in the world, yet less than half of the calories produced are converted to food owing to the dominance of oil palm cultivation [46].

Management of soil nutrients is a key determinant of the productivity of agricultural lands. Over-exploitation of soil nutrients via harvest without sufficient replacement via fertilizer inputs, lack of cover cropping, compaction from heavy machinery and salinization associated with irrigation are common drivers of soil degradation and decreased agricultural productivity [11,23]. Nutrient depletion alone affects more than 130 million hectares of agricultural lands, about 8% of the global cropland area and over 25% of farmland in Latin America [23]. Approximately 49% of food grown globally is done so outside existing planetary boundaries (*sensu* [47]), with 25% of this coming from the over-exploitation of soil N resources [48].

Technological advances in fertilizer production, pesticides and genetically modified cultivar development have increased the productivity of croplands for land managers with economic means, albeit at the expense of environmental externalities [49]. When examining the yields of the top globally traded crops, Mueller *et al*. [22] found that nutrient and irrigation management were the main sources of differences in potential yield gaps, while coarse-resolution soil organic C and qualitative soil texture datasets did not explain trends [22]. Fertilizer use has increased by approximately an order of magnitude since 1950, with a current global consumption of 109 Tg N y^−1^ [50,51]. After 1970, most of the increase in fertilizer consumption occurred in emerging economies (e.g. BRICS countries; [50]), which have enhanced their participation in global food trade. The enormous increase in N introduced to the world, and the decoupling of N from ecological processes [52], have wreaked havoc on water, soil and atmospheric chemistry [53–56].

Like N, soil P is critical for plant growth and rates of agricultural P fertilization have increased dramatically at a global scale [54]. Soil P bioavailability has received less attention, even though it may limit crop productivity by 20–50% in some regions [57]. Phosphorus often has a limited window of bioavailability in soils, particularly in the acidic soils of the tropics; crop species with growth strategies designed to extract soil P may be more successful in these regions [58]. With the increased use of irrigation and inorganic fertilizers to overcome soil nutrient and water limitations, agricultural yields, and thus the food system, have become increasingly decoupled from local soil biogeochemical systems.

### (c) The role of soil characteristics in the human nutrition of food and feed

It has long been known that soils play a primary role in human nutrition. References to the importance of soil quality for human health appear in religious texts as far back as 1400 BCE [2]. Soils differ greatly in their inherent nutrient availability and thus their ability to impart nutrition to food. This is apparent when examining regional and global patterns in the prevalence of undernourishment. In 2019, approximately 9% of the global population, or 690 million people, suffered from undernourishment [59], and food insecurity and associated patterns in poor nutrition have been increasing at a global scale since 2014.

While there are several causes of undernourishment and food insecurity (e.g. extreme climate events, socio-political and economic forces), some of the world's nutritional insufficiency originates with soil nutrient limitation. The increase in food production over the past 50 years has led to a decrease in some nutritional deficiencies, but Ca, Fe, vitamin A and Zn are still broadly deficient [60]. Micronutrients are of particular concern. The global expansion of food production has increased rates of soil micronutrient mining that can lead to eventual declines in crop yields [61]. This pattern is mainly observed in lower income countries where fertilizers are often economically inaccessible [62]. For example, human Zn deficiency is correlated with soil Zn deficiency in sub-Saharan Africa, some areas of South America and South and Southeast Asia [63,64]. Micronutrient deficiency is one of the leading contributors to global disease burden, affecting approximately 50% of the world's population [65]. Low levels of soil Zn, Cu and Mn, in particular, have been linked to increased child mortality [66]. Micronutrient deficiencies are most common in regions where grains have low nutritional content and are a dietary staple [67], and are more likely to occur in regions with dependence on native pollinators (i.e. vitamin A in South Asia and Fe in sub-Saharan Africa) and with the disruption of plant-pollinator processes [68].

Climate change can also limit the ability of soil to provide essential nutrients to food and feed. For example, crops grown under elevated CO_2_ had 3–17% lower Zn, Fe and protein content. It was particularly notable in C3 staple crops (rice and wheat) and maize (being the only C4 crop affected) [69]. Protein in staple crops and potato is expected to decline by 7.6 and 14.1%, respectively, by 2050 as a result of elevated CO_2_ [70], with the greatest impacts in sub-Saharan Africa and South Asia, including India [70]. Nutritional vulnerability associated with elevated CO_2_ will have a disproportionate impact on the poor, who rely on vegetable food sources for most of their nutritional needs [71].

Soil degradation has been found in most of the world's agricultural land, in both intensive commercial and subsistence agriculture [72], lowering the *per capita* nutritional value of food around the globe [60,73]. Reversing the current trend in declining soil fertility would require building up soil nutrient stocks through practices such as soil liming, soil organic matter addition and micronutrient fortification [61,74,75]. A delineation of soil management zones at regional and global scales in which soil physico-chemical composition is determined spatially would aid the development of specific macro- and micronutrient management strategies for sustainable crop production and soil restoration [76].

## 4. Global patterns in the production and movement of key food and feed crops

### (a) Global patterns in food and feed production

Maize, soy, rice and wheat are the staple crops with the greatest agricultural production globally, providing over 65% of human caloric intake annually [77]. Between 2014 and 2018, these crops accounted for approximately 14 ± 0.1% (698 ± 3.5 million ha) of global agricultural land area. By contrast, grazing lands accounted for approximately 3 billion ha globally, with beef and dairy providing less than 18% of human calorie intake annually [17,78]. To explore global and regional patterns in soil nutrient use and movement in food and feed, we used global estimates of N and P concentrations in crops [79] and regional data on maize, soy, rice, wheat and beef production and export between 2014 and 2018 [17]. We compared patterns in crop production and export with the production and export of beef, which is an important consumer of feed crops at a global scale. We also compared food and feed dynamics with N and P fertilizer use over the period to provide context for N and P transport. For all variables, we used average values over the 5-yr dataset; temporal patterns have been reported elsewhere using longer time series [53,80,81]. When considering import and export patterns, we excluded transport within regions and focused only on large-scale intercontinental transport patterns, thus building on previous work [80].

**Figure 3.**
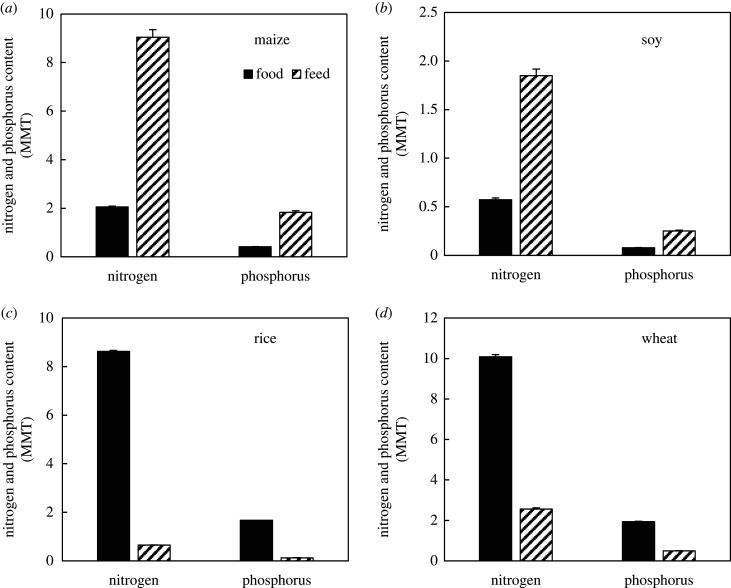
Mean (+s.e.) of global nitrogen and phosphorus content (MMT) in food and feed from 2014 to 2018: (*a*) maize, (*b*) soy, (*c*) rice, (*d*) wheat). Data are from FAOSTAT-3 [37].

Maize and soy are dominant feed crops globally, and are also used for food and other non-food and industrial uses [17]. Approximately 1093 ± 21 million metric tons (MMT) of maize and maize products were grown globally from 2014 to 2018 (table 1). Of the global maize produced over this period, 54 ± 1% was grown for animal feed, while only 12 ± 0.1% was grown for direct human consumption. Assuming a maize N concentration of 15.3 g kg^−1^ N and a P concentration of 3.1 g kg^−1^ P [79], approximately 16.7 ± 0.3 MMT of soil N and 3.4 ± 0.06 MMT of soil P were harvested annually in maize and maize products between 2014 and 2018 (figure 3). North America and Asia were the largest producers of maize globally. Approximately 73 ± 5 and 72 ± 3% of maize production and associated N and P were used for animal feed in North America and Asia, respectively (table 1).

**Table 1.** Production and the mass of feed and food produced for the four main stable crops by region. Values are in million metric tonnes (MMT) for 2014–2018 [37]. Note that feed and food use include crops grown in the region as well as imports.

production	maize		rice		soy		wheat	
	mean	s.e.	mean	s.e.	mean	s.e.	mean	s.e.
Africa	76	2.5	30	0.5	2.9	0.2	26	1.1
Asia	348	11	685	6.6	27	1.3	325	2.9
C. America	30	1.0	1.3	0.0	0.5	0.03	3.5	0.2
Europe	118	4.9	4.1	0.1	10	0.6	255	5.1
N. America	384	11	9.4	0.4	121	3.3	85	2.8
Oceania	0.6	0.02	0.7	0.1	0.04	0.01	25	1.9
S. America	136	7.3	25	0.4	172	4.8	24	1.3
**Feed**								
Africa	27	0.6	2.3	0.04	0.7	0.1	6.4	0.6
Asia	250	17	40	0.45	12	0.7	41	1.1
C. America	19	1.1	0.01	0.004	1.3	0.06	0.1	0.01
Europe	77	2.9	0.5	0.02	2.5	0.20	64	3.0
N. America	145	1.4	0.3	0.03	3.4	0.72	5.9	0.8
Oceania	0.5	0.04	0.005	0.002	0.003	0.001	4.3	0.4
S. America	72	4.0	0.6	0.04	4.4	0.60	1.3	0.1
**Food**								
Africa	49	0.8	38	0.6	0.6	0.03	55	1.0
Asia	45	1.2	507	2.0	8.8	0.3	286	3.5
C. America	18	0.1	2.7	0.04	0.02	0.002	6	0.1
Europe	6	0.12	4.9	0.1	0.2	0.01	84	0.6
N. America	5	0.03	4.0	0.04	0.08	0.001	29	0.2
Oceania	0.1	0.00	0.5	0.02	0.03	0.001	2	0.03
S. America	12	0.1	18	0.3	0.09	0.005	24	0.4

The global production of soy from 2014 to 2018 was 333 ± 8 MMT (table 1), with approximately 90% grown in North and South America (averaging 121 ± 3.3 and 172 ± 4.8 MMT, respectively). Soy, a N-fixing plant, had the highest N concentrations among staple crops with 58.6 g kg^−1^ N for food soy and 76.2 g kg^−1^ N for soy cake, a common feed product [79]. Soy also had high P concentrations (7.9 g kg^−1^ P for food soy and 10.3 g kg^−1^ P for soy cake), derived from high P fertilizer use [82]. In the Americas and Europe, over 90% of the soy grown was used for animal feed, accounting for an average of 0.85 ± 0.09 MMT N and 0.12 ± 0.01 MMT P removed from soils (figure 3). In Africa and Asia, similar amounts of soy were used for food and feed, while in Oceania over 90% of the soy produced was used for direct human consumption. Globally, maize and soy feed production concentrated 11 ± 0.3 MMT N and 2 ± 0.06 MMT P annually between 2014 and 2018. For comparison, global beef production, which concentrates vast soil and water resources [83], averaged 69 ± 1 MMT of production, and 5.2 ± 0.05 MMT N and 0.7 ± 0.007 MMT P from 2014 to 2018.

Unlike maize and soy, most of the global rice and wheat production was used for food. Less than 10% of rice production was used for animal feed (table 1). Asia was the largest producer of rice globally, with 685 ± 6 MMT grown from 2014 to 2018, a value that was two orders of magnitude larger than all regions except Africa and South America (table 1). Rice production in Asia concentrated 11 ± 0.1 MMT N and 2 ± 0.02 MMT P in rice harvest over the period. Periodic flooding in rice agriculture can result in large gaseous N losses [84], decreasing the fertilizer use efficiency and contributing to climate change. Wheat production was also primarily used for food, except in Europe and Oceania, where 43 ± 1% and 64 ± 2% was used for feed, respectively (table 1). Wheat had relatively high N and P concentrations (20.8 g kg^−1^ N and 4.0 g kg^−1^ P), but still much lower than soy.

Global productivity of rice, wheat, soy and maize croplands tended to be greater in regions with higher inherent concentrations of soil N (figure 1 and table 1; [42,43]). A major exception to the pattern of soil N-driven productivity was in eastern Asia and Indonesia, where food and feed crop productivity were greater despite relatively low inherent soil N concentrations [85]. The high productivity despite low N concentrations may in large part be owing to the extensive use of organic and inorganic N fertilizers [86].

Though soils with high nutrient contents are most intensively cultivated with globally traded crops, food production is not limited to these soils. For example, the weathered dryland tropical soils of northern Venezuela are not well-suited for wheat production, yet when cultivated with native succulent, dryland-evolved fruit trees, the combination of local climate, edaphic conditions and crop physiology can lead to both higher yields and soil sustainability [87]. Where climate and soil N are unsuitable for cultivation of crops, rangelands provide vast areas of grass feed for livestock [88]. Livestock grown on grass versus feed crops require more land area per unit of food produced and emit slightly more greenhouse gases, primarily because of the longer life cycle of the animals [89]. However, feeding livestock on rangelands increases the availability of grains for human consumption by almost 50% [90].

A small proportion of the nutrients imbedded in food crops becomes biologically immobilized for days to years, while the vast majority quickly become part of the waste stream [53–55]. For example, the loss and waste of food meant for human consumption amounted to 1.6 Gt in 2011 [91], containing an estimated 2.7 MMT of plant-derived N [92]. The FAO estimated that 13.8% of food production was lost in 2016, with the highest rates (20.7%) found in Central and Southeast Asia [93].

### (b) The global movement of soil nutrients via food and feed export

Soil contributions to food and feed span many spatial scales from less than 1 km for food and feed produced locally to over 10 000 km for transcontinental international trade. International trade in food and feed changed dramatically during the latter half of the twentieth century and into the beginning of the twenty-first century [50], resulting in the large-scale displacement of soil nutrients accumulated in crops. Between 1961 and 2009, global crop trade increased the movement of vegetable and animal proteins by a factor of 7.5 and 10, respectively [80]. Livestock accounted for approximately 75% of global crop production in 2009, with China, North America, Europe and Oceania as the primary consumers; animal feed constituted the largest contribution to exported N [80]. Nitrogen imbedded in animal production increased by 60% from 1986 to 2009, while the increase in per capita N ingestion and consumption of animal protein both increased by only 12% [94]. Between 1960 and 2011, imbedded P in globally traded food and feed increased 7.5 times, which led to an expansion of agricultural P flows for human food (28%), animal feed (44%), and other crops (11%) [54]. Most of the soil P transported via food and feed trade originated from the Americas. Net P imports occurred in regions with large populations (Asia), large livestock production (Europe) or with intense food security challenges (Mediterranean basin) [54].

**Figure 4.**
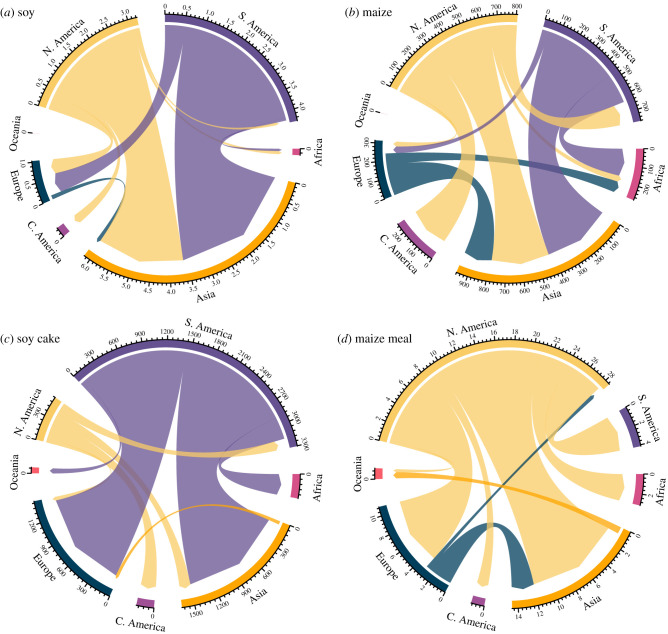
Mean annual intercontinental N export and imports of (*a*) total soy, (*b*) total maize, (*c*) soy cake, and (*d*) maize meal over a 5-yr period from 2014 to 2018. Soy cake and maize meal are primarily used for livestock feed. Colours distinguish region and width of arrow indicates size of export; arrow direction symbolizes export direction. Data from the Food and Agriculture Organization [17]. Only exports greater than a minimum threshold of 0.75% of the sum of all region exports are included in figure. (Online version in colour.)

The four key staple crops, maize, soy, rice and wheat, are widely traded and exported out-of-region, transporting the calories and nutrients imbedded within the harvested crops. From 2014 to 2018, North and South America were the largest exporters of soy and maize crops (figure 4*a,b*, electronic supplementary material, figure S1a,b, table 2). Soy exports resulted in the intercontinental transport of 3.2 ± 0.1 MMT N y^−1^ and 0.4 ± 0.02 MMT P y^−1^ from North America. South America exported 4.3 ± 0.3 MMT N y^−1^ and 0.6 ± 0.05 MMT P y^−1^ in soy. For context, this was equivalent to 58% and 23% of the total agricultural N fertilizer use on all crops in South and North America, respectively, over the same interval. The high N concentrations in soy derive at least in part from biological N-fixation, but N export from these high producing regions illustrates the loss of potential soil nutrients. Phosphorus export in soy from these regions was also high, equivalent to approximately 9% of the total P fertilizer used in these regions (table 2). During the 5-yr period, 14 ± 1% and 58 ± 4% of soy exported from North and South America, respectively, was used for animal feed. South America strongly dominated the soy cake export market for animal feed, moving 3.2 ± 0.1 MMT of soil N y^−1^ and 0.43 ± 0.01 MMT of soil P y^−1^ around the world (note that nutrient concentrations were higher for soy cake than for unprocessed soy [79]).

**Table 2.** Mean and standard errors of mass (MMT), nitrogen (KT) and phosphorus (KT) content of exported rice, wheat, soy and maize between 2014 and 2018. Values exclude export within regions. Export values are compared with total nitrogen and phosphorus fertilizer use (both in MMT) for all of agriculture in each region during the period.

	rice		wheat		soy		maize		sum		fertilizer	
export	mean	s.e.	mean	s.e.	mean	s.e.	mean	s.e.	mean	s.e.	mean	s.e.
mass (MMT)												
Asia	14.74	0.50	0.72	0.06	0.33	0.02	0.10	0.01	15.88	0.50		
Europe	0.36	0.01	67.48	2.79	2.17	0.28	16.05	0.53	86.06	3.26		
Africa	0.11	0.05	0.02	0.01	0.12	0.04	0.79	0.30	1.03	0.28		
S. America	1.36	0.11	4.07	1.21	72.72	5.42	42.68	2.68	120.83	8.38		
C. America	0.05	0.02	0.96	0.18	0.00	0.00	1.05	0.26	2.06	0.30		
N. America	2.40	0.12	42.53	1.01	55.07	2.23	53.31	4.26	153.31	4.45		
Oceania	<0.01	<0.01	15.84	1.30	<0.01	<0.01	0.07	0.01	15.91	1.31		
crop nitrogen (KT)											nitrogen (MMT)	
Asia	221.14	7.43	14.90	1.31	19.12	1.43	1.51	0.20	256.67	7.81	63.99	0.25
Europe	5.41	0.22	1403.59	58.13	127.28	16.30	245.54	8.18	1781.82	75.09	14.74	0.11
Africa	1.63	0.79	0.36	0.13	7.17	2.23	12.03	4.63	21.19	5.34	3.88	0.18
S. America	20.44	1.58	84.56	25.13	4261.57	317.62	653.00	41.00	5019.56	363.06	7.29	0.41
C. America	0.76	0.31	20.00	3.65	0.13	0.07	16.04	4.00	36.93	5.07	1.73	0.13
N. America	36.02	1.73	884.71	21.08	3226.81	130.87	815.65	65.17	4963.19	141.56	14.34	0.06
Oceania	<0.01	<0.01	329.37	27.03	0.20	0.01	1.14	0.17	330.72	27.15	1.86	0.05
crop phosphorus (KT)											phosphorus (MMT)	
Asia	42.75	1.44	2.86	0.25	2.58	0.19	0.31	0.04	48.50	1.49	24.56	1.03
Europe	1.05	0.04	269.92	11.18	17.16	2.20	49.75	1.66	337.88	13.68	3.73	0.06
Africa	0.32	0.15	0.07	0.03	0.97	0.30	2.44	0.94	3.79	0.98	1.47	0.05
S. America	3.95	0.30	16.26	4.83	574.51	42.82	132.31	8.31	727.03	52.08	6.37	0.20
C. America	0.15	0.06	3.85	0.70	0.02	0.01	3.25	0.81	7.26	1.01	0.73	0.08
N. America	6.96	0.33	170.14	4.05	435.01	17.64	165.26	13.20	777.38	20.99	5.06	0.06
Oceania	<0.01	<0.01	63.34	5.20	0.03	<0.01	0.23	0.04	63.60	5.22	1.24	0.02

While soy cultivation originated in China, Chinese farmers focused on maize and rice during the twentieth century owing to higher net economic returns per hectare on crops other than soy [80]. The combination of changes in Chinese domestic and foreign policies in the 1990s and rising living standards in China prompted rapid growth in soy feed and oil consumption from virtually no soy imports in 1990 to comprising up to 65% of global soy imports in 2018 [93]. Brazil's expanding land availability, ability to double-crop soy with maize in its tropical climate, and USA–China trade-tariff policies propelled Brazil's soy production from 10 million ha in 1980 to 35 million ha in 2017. This growth has primarily occurred on nutrient-poor Oxisols of the Cerrado region through economic and subsidy assistance, use of fertilizers and liming, and development of suitable cultivars. At the same time, the decline of soy production in China and its substitution with N-intensive crops have led to a shift in the N balance from negative (soy) to largely positive, with almost half of the contribution (49%) related to cropland conversion and increased mineral N fertilizer application (51%) [95].

Annual N and P transport of maize was an order of magnitude lower than soy from these regions, with average maize exports displacing 0.82 ± 0.07 MMT N y^−1^ and 0.17 ± 0.01 MMT P y^−1^ from North American soils between 2014 and 2018, and 0.65 ± 0.04 MMT y^−1^ and 0.13 ± 0.01 MMT P y^−1^ from South American soil. Maize export was equivalent to approximately 6% and 9% of the regional-scale total N fertilizer usage for North and South America, respectively. Asia was the largest importer of these embedded nutrients (figure 4*a,b*, electronic supplementary material, figure S1a,b). Animal feed only accounted for 3.5 ± 0. 2% and less than 0. 1% of maize exports from North and South America, respectively, predominantly as maize meal.

**Figure 5.**
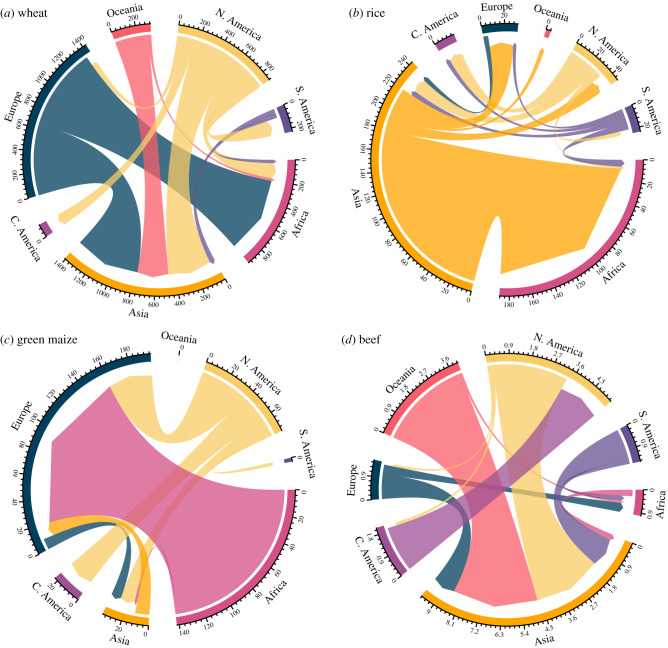
Mean annual intercontinental N export and imports of (*a*) wheat, (*b*) rice, (*c*) green maize and (*d*) beef over a 5-yr period from 2014 to 2018. These are all dominant food products. Colours distinguish region and width of arrow indicates size of export; arrow direction symbolizes export direction. Data from the Food and Agriculture Organization [17]. Only exports greater than a minimum threshold of 0.75% of the sum of all region exports are included in figure. (Online version in colour.)

Asia and Europe were the largest importers of soy cake and maize meal for animal feed (figure 4*c,d*, electronic supplementary material, figure S1c,d). Combining the export of soy cake and maize meal with the embedded nutrients in the export of cattle meat accounted for a total intercontinental movement of approximately 3.9 ± 0.1 MMT N y^−1^ and 0.53 ± 0.01 MMT P y^−1^, a value equivalent to the total N fertilizer use in Africa and 43% of the total P fertilizer used in Oceania (table 2). Global export of cattle meat embedded only 0.33% of the N and 0.15% of P y^−1^ exported as soy cake and maize meal at a global scale. Oceania had the highest soil nutrient export embedded in cattle meat at 4.1 ± 0.2 KT N y^−1^ and 0.2 ± 0.01 KT P y^−1^ (figure 5, electronic supplementary material, figure S2).

The imbedded soil nutrients in the global transport of wheat originated primarily in Europe (1.4 ± 0.06 MMT N and 0.27 ± 0.01 MMT P) and North America (0.88 ± 0.02 MMT N and 0.17 ± 0.004 MMT P) from 2014 to 2018. Most of this wheat was destined for Asia and Africa (figure 5*a*, electronic supplementary material, figure S2a). Rice crops and their associated nutrients were largely produced in Asia, with Africa as the dominant importing region. Africa in turn exported the majority of world's green maize (a food product), which was sent to Europe (figure 5*b*; electronic supplementary material, figure S2b).

## 5. Small-scale food and feed production

Small farms account approximately 40% of the world's agricultural land [96]. Of these, organic agriculture was estimated to account for approximately 70 million ha globally, or 1.4% of the global agricultural land area in 2017 [97]. Small and diverse farmlands are responsible for the provision of most global micronutrients and protein sources for human consumption (53–81% and 57%, respectively; [98]). Small farms provide 51% of global crop production, with very small holdings (less than 2 ha) responsible for 30–34% of the food supply on 24% of gross agricultural area [99]. Intensification of traditional agriculture will be required to satisfy the growing demand for food. However, crop yields have historically been significantly lower in organic and/or family-based traditional agriculture (OFBTA) than in conventional agriculture [100–102]. It is notable that multi-cropping and crop rotations may be able to narrow the yield gap [103].

There are many co-benefits to OFBTA for soil and its relationship to people. For example, OFBTA tends to have higher agricultural landscape diversity than large-scale cropping systems [98,99]. Greater landscape diversity in turn can contribute to higher genetic diversity and enhanced adaptability to a wider range of edaphic and agro-ecological conditions (*sensu* [104]). Soils under OFBTA encompass both cultural and management principles [105] that have the potential to promote characteristics of soil sustainability (i.e. lower erosion rates [106,107]; greater microbial biomass [108], improved soil quality indicators [100], and lower reactive N soil losses and new N additions [109]), as well as providing a local and culturally relevant source of food and feed.

Traditional agriculture in the tropics represents approximately 20% of the global tropical crop area [110] and encompasses a variety of shifting cultivation types primarily performed as part of subsistence agriculture. Slash and burn agriculture is the oldest and most widely used shifting cultivation in the tropics, where fire ash provides soils with base cations and P, reduces in bulk density, and increases pH. However, burning also releases C, N, sulfur and micronutrients to the atmosphere, resulting in a net loss of C and N from soils. Burning can also reduce microbial biomass and result in the loss of soil fauna and their contribution to soil ecosystem functioning [111,112]. Land-intensification derived from political and socio-economic pressures has resulted in shorter fallow periods and reduced the resilience of successional ecosystems and their soil potential to contribute to food and feed provisions [113]. Land-intensification has resulted in a transformation of traditional indigenous soil management practices in some regions [114,115]. Fire-free alternatives that improve soil quality have been proposed, such as slash and mulch [116], mixed crop-livestock systems [117] and ash and compost addition [118]. The incorporation of indigenous soil knowledge [119], broadening agro-ecological perceptions (e.g. termite management for soil fertility [120]) and the implementation of government-led environmental policies for sustainable soil management practices could empower local communities and contribute to food security, enable food sovereignty and contribute to the preservation of cultural heritage.

## 6. Soil contributions to food security for a growing population

Current trends in global population growth will require a 70–100% increase in food production by 2050 [121]. Several soil management approaches have been proposed to address the increased need for food. These include shifts to more nutrient-efficient diets [121], strategic intensification and technological improvements [29], restoration and maintenance of soil fertility and stability [122] and enhancing resilience in the face of climate change [5,123].

Feed crops currently account for almost 40% of global agricultural production, including the use of some of the most fertile soils in the world. In the USA alone, more than half (60%) of agricultural production is used for animal feed [31]. From a soil management perspective, animal feed-based agriculture is an inefficient use of soil resources to feed human populations as less than 10% of the calories and protein from feed is subsequently consumed by humans [124]. Beef production alone uses 10 times more cropland than the production of food crops with an equivalent calorie and protein content [125]. Livestock remains an important source of protein and nutrition in many regions of the world, but also represents a drain on potential food calories when animals are fed with crops that can also be used directly as food for people [121]. A shift in calorie source from animal to more plant-based foods is a dietary change that encompasses more than just agricultural management, but also a complex set of socio-economic and cultural issues [126].

Strategic intensification and technology improvements to reduce yield gaps of existing croplands can also help address growing food demand [29]. Planting crops with genetically improved traits has and will continue to add to increased crop yields [31]. Optimized management in the context of a particular crop, climate and soil type has been shown to reduce pollution and waste and increase yields, though the expense of research to understand the ecological context may be an economic barrier [127]. Arable land scarcity can be reduced by strengthening Diversified Farming Systems (DFS), defined as farming practices and landscapes that intentionally include functional biodiversity at multiple spatial–temporal scales to preserve nature's contributions to people from which agriculture, soil fertility, pest and disease control, water use efficiency and pollination are sustained [128]. When possible, DFS should target selected degraded lands, managed forests or abandoned agricultural areas that are not adjacent to natural ecosystems to minimize their potential vulnerability [129,130].

Maintaining cropland area and long-term fertility of soil through optimal management is a key component of the contribution of soil to future food security. Tens of millions of hectares of agricultural land are lost per year through erosion and poor water management, as well as urbanization and associated development [35,131–133]. Of the total projected new urban expansion, 50–63% is expected to occur in cropland area, which alone would reduce global food production by 1–4% [133]. Reversing the current trend in soil fertility loss would require the rebuilding of soil nutrient stocks. A suite of practices such as soil liming, organic matter amendments [134,135] and micronutrient fortification [61,74,75] are examples of practices that have been deployed. Enhancing soil organic matter content has also been proposed as a means to rehabilitate nutrient retention in soils. Increased soil organic matter stocks not only promotes higher yields and soil quality but also provides a co-benefit for climate change mitigation through increased soil C storage and decreased greenhouse gas emissions relative to other fertilizers [5,135–137].

Harvesting food moves C and nutrients from soils to areas where humans and sewage are concentrated. Farming practices that recycle, replace or restore these nutrients can reduce the mining effect of harvest and enhance the longevity of soils for food production. Soil quality assessment approaches have shown that soil biological, chemical and physical indicators improve with practices including organic matter additions, conservation-tillage practices, crop rotation and organic agriculture [100]. Planting leguminous cover crops is a traditional and widely used method of increasing soil N content [138]. When applied to appropriate soil types, soil amendments including biochar and compost can be used to improve soil fertility. Compost amendments have been shown to increase crop yields, improve soil microbial diversity and stimulate mycorrhiza formation [135,139].

## 7. Conclusion

The combination of population growth, soil degradation, shifting diets and climate change pose significant challenges for the future of global food and feed production. Variation in inherent soil properties and climate provide a diverse template of environmental conditions for the provision of food and feed and contribute to global-scale patterns in crop production, the nutritional quality of food and feed produced, and the ultimate impact on soils. The harvest and transport of food and feed mine soils of essential nutrients. Fertilizers are rarely able to fully replace the nutrients that are removed, and thus over time, repeated harvests contribute to soil degradation. Where economically feasible, fertilizers and irrigation can extend the productive capacity of soils, but often at significant environmental and economic costs.

Global-scale analyses of the production of key grain crops showed that soil resources are increasingly being concentrated in the production of animal feed at the expense of the direct provision of food for growing human populations. Feed production exhibited large inefficiencies with regard to important nutrients like N and P. For example, beef cattle concentrated only half of the soil N and P harvested from crops that were processed into maize meal and soy cake. Global trade in the dominant grain crops and their associated nutrients extracted from soils is growing globally. Analyses of recent trade patterns revealed large-scale exports and imports of soy, maize, wheat and rice and their associated nutrients across intercontinental trade routes, resulting in the displacement of millions of tonnes of soil N and P annually. Much of the nutrients and biomass transported were subsequently concentrated in waste streams.

Small-scale agriculture contributes significantly to the production of food and feed for the global population and may retain a higher proportion of the soil nutrient stock at a local scale. However, significant yield gaps will need to be overcome via intensification or other approaches to keep up with growing food demand. There are several proposed approaches to address the increased need for food and feed. These include shifts to more nutrient-efficient diets [121], strategic intensification and technological improvement [29], restoration and maintenance of soil fertility and stability [122] and enhancing resilience in the face of global change [5,123]. These and other innovative approaches will be needed to sustain the contribution of soils to the provision of food and feed into the future.

## Data accessibility

This article has no additional data.
